# A Noval Approach of Altered Cast Technique in Bilateral Partial Maxillectomy Patient with Severely Restricted Mouth Opening

**DOI:** 10.5402/2011/607175

**Published:** 2010-10-31

**Authors:** Shuchi Tripathi, Pooran Chand, Raghuwar D. Singh, Ramashankar Siddharth, Balendra P. Singh

**Affiliations:** Department of Prosthodontics, Faculty of Dental Sciences, C.S.M. Medical University (Upgraded K.G.M.C.), Lucknow, Uttar Pradesh 226003, India

## Abstract

Patients with extensive head and neck injuries due to trauma and/or extensive surgical procedures often exhibit a severely limited ability to open the mouth. For the dentist involved in prosthodontic treatment of such patients, restricted mouth opening commonly leads to compromised impressions and prostheses especially in cases where both hard and soft palatal parts have been resected. In prosthodontic treatment, the loaded impression tray is the largest item requiring intraoral placement. During impression procedures, wide mouth opening is required for proper tray insertion and alignment. This is not possible in patients with restricted mouth opening, so a modification of the standard impression procedure is often necessary to accomplish this fundamental step in the fabrication of a successful prosthesis. An alteration in the final impression procedure was made using altered cast technique for fabricating an obturator prosthesis with soft palate extension, and the result obtained was quite satisfactory.

## 1. Introduction

Postsurgical maxillary defects predispose the patient to hypernasal speech, liquid leakage into the nasal cavity, and impaired masticatory function. In the total rehabilitation of the maxillectomy patient, the Maxillofacial Prosthodontist has two primary objectives; first to restore the functions of mastication, deglutition, and speech and secondly to achieve normal orofacial appearance [1]. Ideally, any anatomic defect should be surgically reconstructed. However, when surgical reconstruction is contraindicated, prosthetic reconstruction must be employed to restore anatomy, function, and esthetics. The goals of prosthetic rehabilitation for total and partial maxillectomy patients include separation of oral and nasal cavities to allow adequate deglutition and articulation, possible support of the orbital contents to prevent enophthalmos and diplopia, support of the soft tissue to restore the midfacial contour, and an acceptable aesthetic results [2].

Limited mouth opening often complicates and compromises the treatment of patients, who underwent maxillectomy due to combined effect of radiotherapy and surgical procedures done. Trismus may occur due to inflammation and insults to muscles of mastication and other surrounding tissues [3]. A method of overcoming impression difficulties that uses a custom impression tray modified with altered impression for such patients is outlined and illustrated in present case report to simplify the impression procedure to obtain a successful prosthesis.

## 2. Case Report

**Figure 1:**
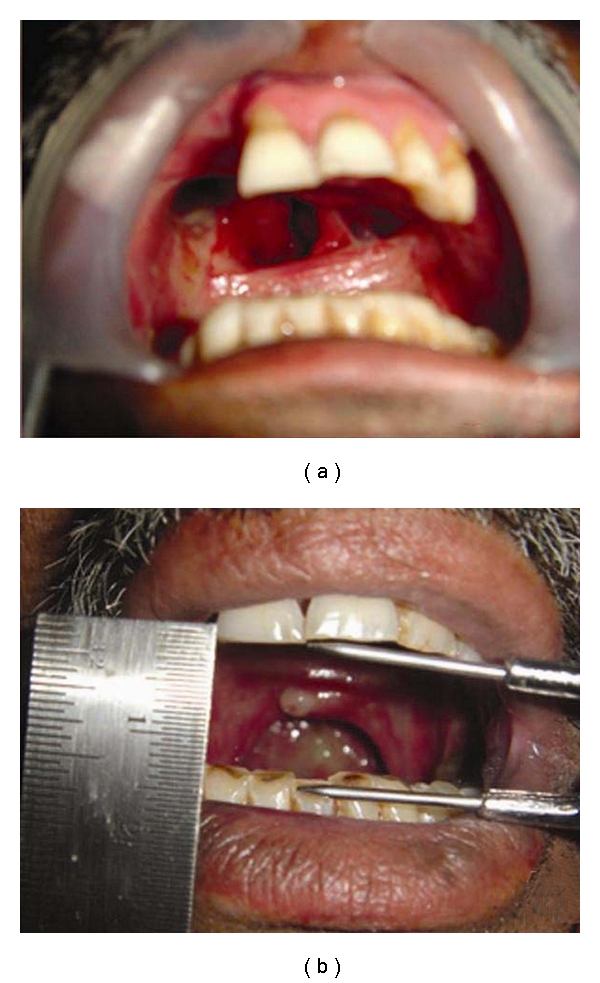
Intraoral view of the patient.

A 49-year-old male patient presented with a chief complaint of missing teeth in the upper jaw. His primary concerns were inability to chew food, difficulty in speech, and the regurgitation of the food into the nasal cavity. He had been diagnosed with Mucoepidermoid carcinoma of minor salivary glands for which a bilateral partial maxillectomy was done, leaving a small portion of the premaxilla. Resection was followed by postsurgical radiation therapy. No preprosthetic consideration was made before surgery, and this probably had worsened the case resulting in irretrievable limited mouth opening. Intraoral examination revealed bilateral missing maxillary posterior teeth and teeth present in premaxilla were 11, 21, 22, and 23 (Figure 1(a)). Oral hygiene was poor and teeth were periodontally affected with grade I mobility. Reduced mouth opening of 1.5 cm was noticed (Figure 1(b)). A part of the soft palate which was in the continuation with hard palate was also resected, due to which functional inefficiency of soft palate was seen. Obturator prosthesis with soft palatal extension was planned for covering the defect improving speech, mastication, and esthetics. 

**Figure 2:**
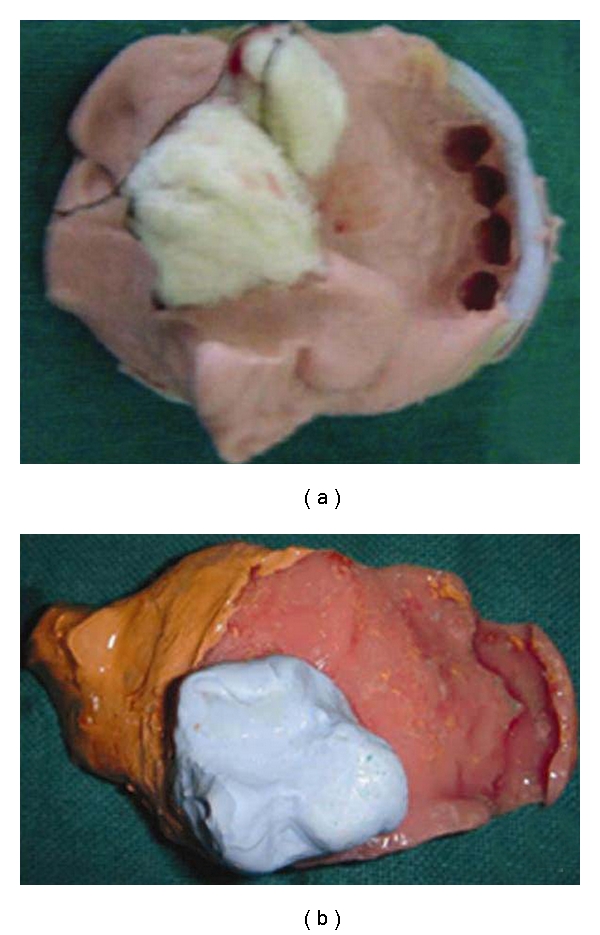
Preliminary and final impression of the maxillary arch.

**Figure 3:**
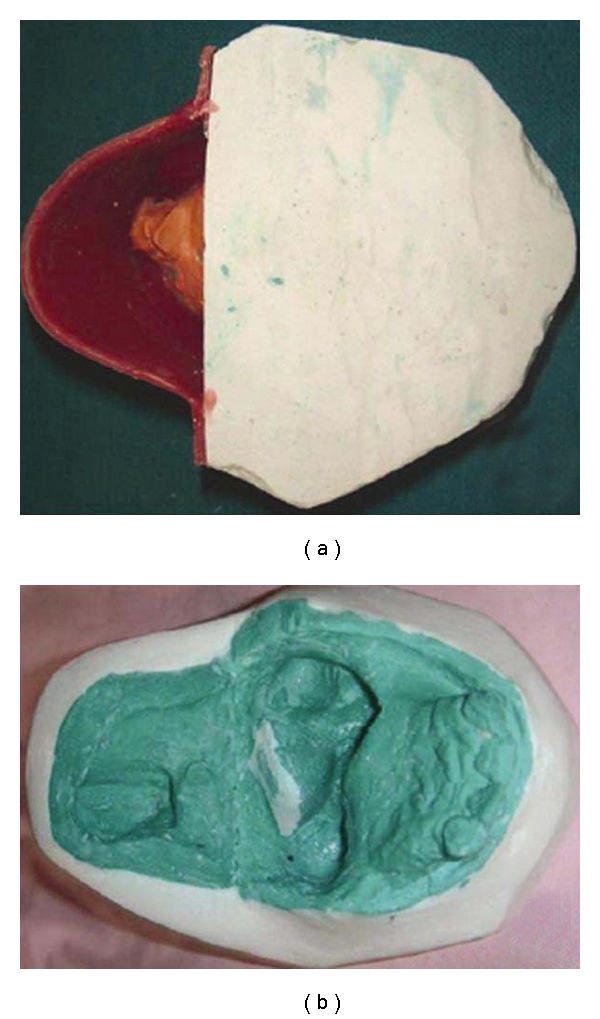
Final cast of the defect.

Oral prophylaxis was completed and a primary impression of the hard palate was made by an irreversible hydrocolloid impression material (Zelgan, Dentsply India Pvt Ltd, India) with modifying the stock tray in the defect region (Figure 2(a)). The impression was then poured in Type III dental stone (Kalstone, Kalabhai, Karson Pvt Ltd, Mumbai, India). A special tray was then constructed following a predetermined outline on the stone model using autopolymerizing acrylic resin (Dental Products of India Ltd, New Delhi, India). The bulb portion of the hard palate was made with putty silicon impression material (3M ESPE AG, Germany) and adhered to the custom tray. A posterior extension of medium fusing impression compound (Y-Dents impression compound; MDM Corporation, New Delhi, India) was added to acrylic tray to contact the pharyngeal wall when it closes during speech and swallowing. It was confirmed that the extensions was made at the level of the hard palate and at the level of the most active movement of the pharyngeal sphincter. Right and left neck movement and swallowing was performed by the patient for recording the soft palate extension. The patient was asked to perform the same movements during the final wash impression of soft palate with zinc-oxide eugenol impression paste (DPI Impression paste, Dental products of India, Mumbai, India), after scraping the impression compound about 0.5 mm (Figure 2(b)). This impression was placed over the stone model of hard palate, in which the posterior landmark area was trimmed for properly joining the extended impression. Now proper beading and boxing of the soft palatal extended part was performed (Figure 3(a)) for making the master cast by Altered cast technique (Figure 3(b)). Jaw relation was recorded at reduced vertical height for providing better comfort to tissues and also for ease in insertion and removal of the prosthesis. Teeth arrangement was done and prosthesis was finally verified esthetically and physiologically during try-in procedure.

**Figure 4:**
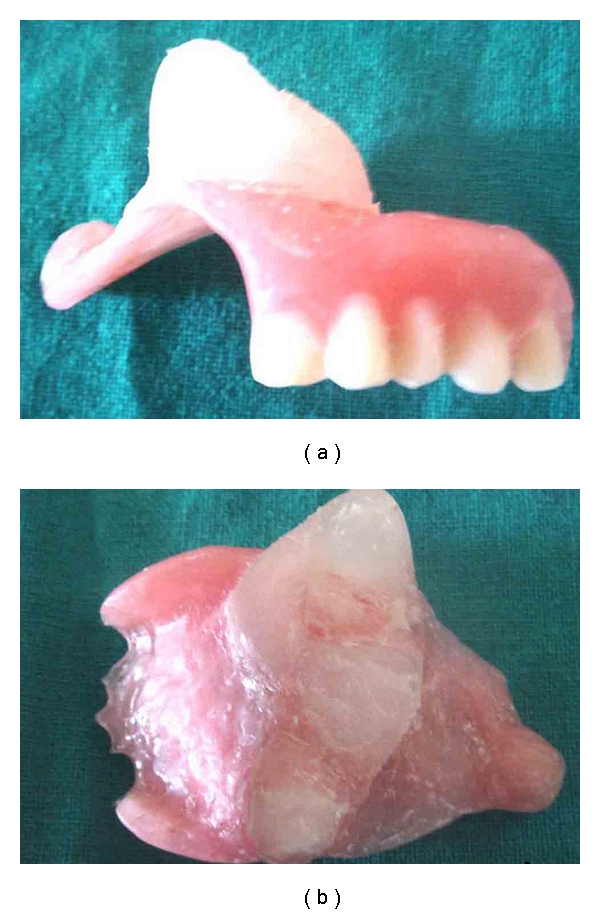
Final Prosthesis.

**Figure 5:**
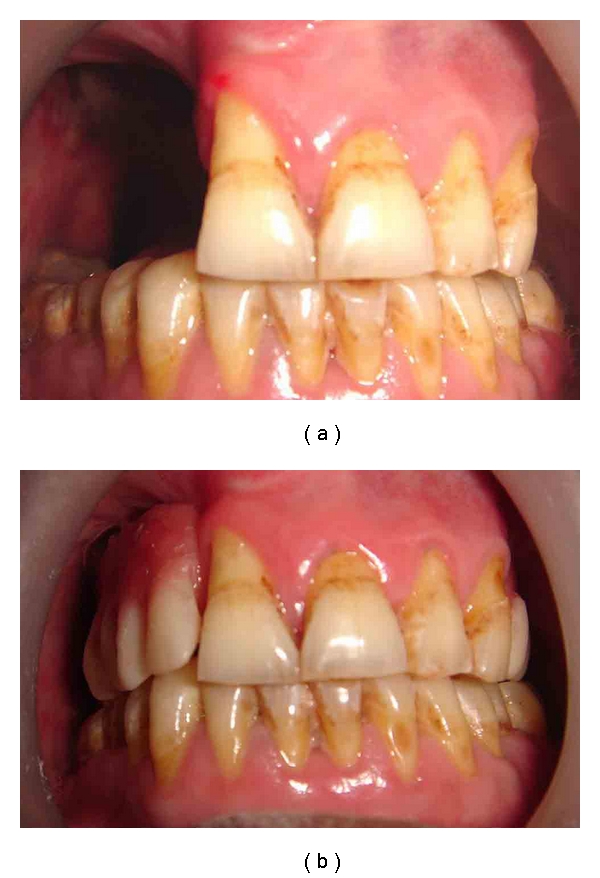
Pretreatment and posttreatment intraoral view of the patient.

As there was restricted mouth opening, height of the bulbs was reduced for curing with heat-cured acrylic resin (Trevalon Hi, Dentsply India Pvt Ltd, India). After fabrication the prosthesis was finally finished and polished. The tissue surface of the bulb was then finally relined with the soft liner (PermaSoft Denture Liner; Dentsply Austenal, York PA) (Figure 4). The insertion of the obturator into the defect was possible in a rotational path; from mediodistal to bucco-anterior. The obturator was fully seated (Figure 5) and confirmed that the prosthesis was easily placed and removed by the patient. Speech of the patient was then evaluated and reduced hypernasality was noted. Patient was given instructions regarding prosthesis use and regular followup. At the 6 month evaluation of the prosthesis, no further complications were found. The patient was satisfied with the function, esthetics, and retention of the prosthesis and greatly improved oral hygiene and maintenance were observed.

## 3. Discussion

Bilateral complete maxillectomy is a relatively uncommon surgical procedure resulting in devastating effects on the cosmetic, functional, and psychological aspects of a patient's life [4]. As multiple surgeries cannot be performed at this stage, prosthetic restorations have become the preferred method for the rehabilitation of complex mid-facial defects like the bilateral or unilateral maxillectomy. Recently, it has been reported that obturator prosthesis function is closely related to patient quality of life [5]. They allow rapid, single stage reconstruction which is important since improvement in the quality of life is of paramount concern as in many of these patients, surgery may be only palliative [6].

In most of the cases, trismus is exacerbated by surgery prior to radiation and by radiation field that includes the muscles of mastication and the temporomandibular joint. In patients treated for nasopharyngeal carcinoma with radiotherapy, the maximum interincisal distance was reported to be reduced to 97% of its original distance in the first nine weeks after radiotherapy. Further reduction to 73%, 69%, 68%, and 67% were observed in 12, 24, 36, and 48 months, respectively [7]. If not managed at initial days, it will lead to life-long reduced mouth opening, difficulty in eating, and problem in performance of dental procedures. Reason for reduced mouth opening in the present case report was the same, and the patient was not ready for any further surgery for improvement of fibrosis. Exercise could not help the patient for improving mouth opening due to permanent scar formation [8]. As the mouth opening was just 1.5 cm, it was not possible to record soft and hard palatal portion in a single impression so modification in the impression procedure by using altered cast impression technique with some modification was thought. Advantages of the altered cast technique used in the present case report include simplified tray manipulation, decreased patient trauma, the ability to use a custom fabricated tray for optimal impression material thickness, precise intraoral positioning, and stability of the tray. 

 Clinical applications of magnetic attachments in maxillofacial prostheses have been used as overdenture abutments and for attachments between components of the prostheses [8, 9]. Magnet attachments need efficacy for adjustment of final placement of upper and lower parts of the prosthesis during insertion, and also the defect was bilateral and large which needed more complicated treatment. Keeping these points in view a conventional type of obturator with posterior extension in a single piece was thought for convenience of the patient. Mediolaterally the mouth width was competent enough for placement of the prosthesis after slight tilting of the prosthesis. Hence reducing the height of bulbs was an effective option for prosthesis insertion and also the soft liner used was somewhat flexible for helping in placement of prosthesis [10]. 

Insertion and removal of large prostheses used for rehabilitation of maxillectomy requires good neuromotor coordination and an adequate mouth opening. Absence of these factors in the present case report had compromised the prognosis of the treatment. To accomplish this, heights of the bulbs were reduced and to compensate for the compromised direct retention, maximum use of the lateral periphery contour of defect and scar tissue was desired. A maxillofacial prosthesis cannot restore the intricate neuromuscular structure but can only be used as an alternative means for appropriate function. How successful that alternative is, will depend upon the patient's ability to accept the defect and to adapt to an alternative environment.
